# Birth Order and Sibling Sex Ratio of Children and Adolescents Referred to a Gender Identity Service

**DOI:** 10.1371/journal.pone.0090257

**Published:** 2014-03-20

**Authors:** Doug P. VanderLaan, Ray Blanchard, Hayley Wood, Kenneth J. Zucker

**Affiliations:** 1 Gender Identity Service, Child, Youth and Family Services, Centre for Addiction and Mental Health, Toronto, Canada; 2 Department of Psychiatry, University of Toronto, Toronto, Canada; Federal University of Rio de Janeiro, Brazil

## Abstract

In adult male samples, homosexuality is associated with a preponderance of older brothers (i.e., the *fraternal birth order effect*). In several studies comparing gender dysphoric youth, who are likely to be homosexual in adulthood, to clinical or non-clinical control groups, the findings have been consistent with the fraternal birth order effect in males; however, less is known about unique sibship characteristics of gender dysphoric females. The current study investigated birth order and sibling sex ratio in a large sample of children and adolescents referred to the same Gender Identity Service (*N* = 768). Probands were classified as heterosexual males, homosexual males, or homosexual females based on clinical diagnostic information. Groups differed significantly in age and sibship size, and homosexual females were significantly more likely to be only children. Subsequent analyses controlled for age and for sibship size. Compared to heterosexual males, homosexual males had a significant preponderance of older brothers and homosexual females had a significant preponderance of older sisters. Similarly, the older sibling sex ratio of homosexual males showed a significant excess of brothers whereas that of homosexual females showed a significant excess of sisters. Like previous studies of gender dysphoric youth and adults, these findings were consistent with the fraternal birth order effect. In addition, the greater frequency of only children and elevated numbers of older sisters among the homosexual female group adds to a small literature on sibship characteristics of potential relevance to the development of gender identity and sexual orientation in females.

## Introduction

Numerous studies have shown that there is a unique relationship between older brothers and homosexuality in males (for review, see [1]–[3]). Homosexual males tend to have significant preponderances of older brothers. Other categories of siblings (i.e., older sisters, younger brothers, younger sisters), however, do not appear to be uniquely associated with male sexual orientation. The unique relationship between older brothers and male sexual orientation has been termed the *fraternal birth order effect*.

The most prominent hypothesis regarding the fraternal birth order effect posits that maternal-fetal interactions are responsible for this association [4], [5]. Specifically, this effect is hypothesized to reflect a mother’s immune response to the gestation of successive male fetuses. According to the hypothesis, some mothers experience an immune response to male-specific antigens linked to the Y chromosome, an immune response involving the production of anti-male antibodies. This immune response is thought to become more likely with each successive male fetus gestated and increases the probability that the typical action of male-specific antigens in the developing fetal brain will be diminished. This affects neural areas underlying sexual orientation such that matured males exhibit a female-typical sexual partner preference (i.e., sexual partner preference for adult males). Hence, in later-born sons, there is an increased probability of homosexuality. This line of reasoning has been termed the *maternal immune hypothesis*. Although no laboratory evidence directly supporting the maternal immune hypothesis is available, there are three main lines of evidence that indirectly demonstrate that this hypothesis is tenable.

First, evidence indicates that the tenets of the maternal immune hypothesis are plausible (for review, see [2], [3], [5], [6]). Fetal cells routinely enter maternal circulation during pregnancy (e.g., [7]–[11]). Male fetal cells appear to be capable of eliciting a maternal immune response because women who have given birth to sons are more immunologically reactive to certain male-specific proteins than those who have not [12]. Because the fetal blood-brain barrier is not fully developed, maternal anti-male antibodies that are able to pass from the mother into fetal circulation could bind to male-specific antigens on fetal brain cells. Candidate Y-linked antigens that could be targeted by maternal anti-male antibodies and affect fetal neural development of brain areas related to sexual orientation have been identified [2], [13]. Given this information, it is feasible for mothers to be exposed to male-specific antigens and develop an immune reaction to these substances that affects the sexual orientation of their sons.

A second line of evidence is related to the timing of the fraternal birth order effect. In studies examining birth weight, older brothers, and sexual orientation, homosexual men and probably prehomosexual feminine boys who had greater numbers of older brothers also had lower birth weights [13], [14]. Hence, even at the time of birth, there is a physical marker of sexual orientation (i.e., birth weight) that is related to the number of older brothers. Furthermore, in a study by Bogaert, the association between male sexual orientation and biological siblings (i.e., born from the same mother) and non-biological siblings (i.e., adoptive, step, or paternal half-siblings) was examined [15]. Whether and how long probands were reared with these siblings was also considered. Biological older brothers significantly predicted male sexual orientation regardless of whether or how long probands were reared with these brothers. In contrast, the remaining sibling categories, including non-biological older brothers, did not. In line with the maternal immune hypothesis, these findings strongly suggest that the basis of the association between older brothers and male sexual orientation is prenatal in origin.

The majority of research bearing on the plausibility of the maternal immune hypothesis comprises the third line of evidence. Because the maternal immune hypothesis posits mechanisms that have the potential to be active in any situation where a mother gestates successive male fetuses, the plausibility of this hypothesis is contingent on the ubiquity of the fraternal birth order effect. One approach for establishing the ubiquity of this effect has been to examine a variety of sample types. Overwhelming support has been garnered for the maternal immune hypothesis using this approach. In adults, the fraternal birth order effect has been documented in university and community convenience samples, national probability samples, clinical samples of male-to-female transsexuals, clinical samples of men who are primarily attracted to prepubescent or pubescent children, and archival samples of men interviewed decades ago (for review, see [1]–[3]). Importantly, this effect has been documented in several countries, including Canada [4], Italy [16], the Netherlands [17], Samoa [18], Spain [19], the UK [20], and the USA [21].

Samples of children and adolescents represent another important sample type to consider. If the maternal immune hypothesis is correct and the timing of the fraternal birth order effect is the prenatal period, then this effect should be evident even among children who show characteristics that are indicative of future adulthood homosexuality or adolescents who have only recently begun to express an overt homosexual sexual orientation. To date, four studies have reported data indicating that the fraternal birth order effect exists in clinic-based samples of male children and adolescents [17], [22]–[24]. Due to the difficulty of directly assessing sexual orientation in youth, particularly among young children, these studies adopted a strategy of examining sibship composition in relation to probable adult sexual orientation. Gender Dysphoria (known as Gender Identity Disorder prior to DSM-5) manifests as extreme cross-gender behavior and identification [25]. Prospective research has shown that the significant majority (60–80%) of boys referred for Gender Dysphoria exhibit same-sex sexual attraction in adolescence and adulthood in relation to their birth sex [26]–[29] and, therefore, can be considered prehomosexual. Thus, it is possible to investigate the fraternal birth order effect in youth samples by comparing male children and adolescents clinically referred for Gender Dysphoria to groups of control children for whom heterosexuality is the most likely sexual orientation outcome.

Using this strategy, Blanchard et al. identified patterns consistent with the fraternal birth order effect [22]. The 156 (pre)homosexual child and adolescent males in their sample were later born among their siblings compared to a clinical control sample of males matched for age, year of birth, and number of siblings. In addition, the (pre)homosexual sample had an elevated sibling sex ratio of males to females of 141∶100, which was significantly higher than the ratio of 106∶100 that characterizes Western populations [30], [31]. The sibling sex ratio of the clinical controls was 104∶100, which did not differ significantly from the population ratio. Similarly, Zucker et al. examined the sibships of 333 gender dysphoric male youth and found a significantly higher than expected older sibling sex ratio of 151.8∶100, but a younger sibling sex ratio of 114.2∶100 that did not differ significantly from the population ratio [24]. These males were also significantly later born among their brothers than among their sisters. Blanchard et al. also identified significantly late birth order in a small sample of 21 child and adolescent Dutch gender dysphoric male children and adolescents compared to a clinical control group matched for age and sibship size [17]. Most recently, Schagen et al. reported on the sibship compositions of a Dutch sample of 94 peripubertal gender dysphoric males and 875 non-clinical control peripubertal males [23]. Using logistic regression analysis, this study identified the fraternal birth order effect by showing that number of older brothers was a predictor of group, with gender dysphoric males having greater numbers of older brothers. These studies of male youth samples are, therefore, consistent with both the fraternal birth order effect and the maternal immune hypothesis.

As noted by Blanchard and Klassen, thoroughly assessing the maternal immune hypothesis requires consideration of sibship composition in relation to female sexual orientation as well [5]. Females lack the Y-linked proteins (known or potential antigens) necessary to elicit a maternal immune response capable of affecting neural areas underlying sexual orientation. Thus, any findings indicating that increases in biological older brothers contribute to female sexual orientation would challenge the maternal immune hypothesis. There are no effects of older brothers on female sexual orientation in adult samples [32], but only two studies have considered female youth samples. As is the case with males, Gender Dysphoria in female children and adolescents is associated with an increased rate of homosexual or bisexual sexual orientation in relation to birth sex in most studies (60–99%) [28], [29], [33]; Drummond et al. found that 32% of gender dysphoric girls at follow-up reported bisexual or homosexual sexual attraction and although this figure was not as high as in other studies, they noted that this percentage was substantially higher than the rate of same-sex attraction among females in the general population [34].

In a small sample of prepubertal females, gender dysphoric females (*n* = 22) were significantly earlier born compared to control females (*n* = 147) [35]. Schagen et al. compared the sibship compositions of 95 gender dysphoric and 914 non-clinical control peripubertal females in a Dutch sample [23]. The gender dysphoric females had significantly fewer older siblings, younger brothers, and total siblings. In addition, gender dysphoric females were significantly more likely to be only children. Given these findings, there does not appear to be a fraternal birth order effect among gender dysphoric females.

In addition to these clinic-based studies of gender dysphoric youth, two studies have provided an analysis of available sibship and sexual orientation data using information on a large representative sample of adolescents from the general U.S. population [36], [37]. Sibship information consisted of whether participants had one or multiple older brothers, older sisters, younger brothers, and younger sisters, respectively. In a subset of twin and sibling pairs examined, no significant associations between older brothers or older sisters and whether same-sex sexual attraction was reported were found for males or females [36]. In the second study, for males, there was no evidence of an older brother effect, but those who experienced a non-zero level of same-sex attraction were significantly less likely to have older sisters [37]. In other words, this study found that male homosexual attraction is associated with a deficiency of older sisters rather than an excess of older brothers. For females, same-sex sexual behavior and attraction were associated with a significantly lower probability of having an older brother and significantly fewer sisters. The results of these studies conflict with those of gender dysphoric youth.

Yet, there are a number of factors that raise doubt about the significance of these two studies. Although these studies utilized large adolescent samples, the low population base rates of same-sex attraction and behavior resulted in sample sizes that would be considered small for studies of birth order in relation to sexual orientation. In addition, these studies utilized different methods of sexual orientation classification and imprecise measures of sibships (e.g., “one older brother” or “multiple older brothers” rather than the precise number of older brothers), which may have obscured sibling category effects typically found in other studies. Another potential problem concerns sibship sizes of the male sexual orientation groups. Heterosexual males had larger numbers of siblings overall than the homosexual males and this may have obscured the analyses of group differences in older brothers. Thus, an alternative birth order metric that controlled for sibship size may have produced findings consistent with the fraternal birth order effect [38]. Lastly, the findings of these studies are inconsistent with those reported in studies of adult national probability samples, which did find support for the fraternal birth order effect [39], [40]. It is, therefore, important to examine sibship and sexual orientation in additional youth samples to determine whether findings from youth samples are consistent with the fraternal birth order effect for male homosexuality.

The present study adds to this small literature by examining sibship composition in youth samples. Our previously unexamined clinic-based child and adolescent sample was comprised entirely of male and female children and adolescents referred to the same Gender Identity Service. Thus, in addition to providing sibship data on (pre)homosexual gender dysphoric youth, the present study is the first to report sibship data for gender-referred youth who are predominantly opposite-sex attracted. The fact that all probands were recruited from the same source is also a unique feature of the present study that improves upon the use of clinical or non-clinical control groups in previous similar studies. The sample of 768 probands provides the largest number of gender dysphoric youth considered in a single study of sibship composition to date. Although most probands were referred for Gender Dysphoria, the sample also included some probands referred for other reasons (i.e., transvestic fetishism or issues related to a homosexual sexual orientation). These latter probands were included because the primary focus of the study was on sibship composition and sexual orientation, not basis of referral. Three separate groups were examined: (pre)heterosexual males, (pre)homosexual males, and (pre)homosexual females–too few heterosexual female cases were available to include this group in the current study. The sibship compositions of these three groups were examined. The primary aim was to assess whether (pre)homosexual males, but not females, showed a preponderance of older brothers, which would be consistent with the fraternal birth order effect and the maternal immune hypothesis.

## Methods

### Ethics Statement

Birth order information was obtained from patient charts and the need for written informed consent was waived because the data presented here were collected as part of a chart review study approved by the Centre for Addiction and Mental Health Research Ethics Board.

### Participants

The probands were 210 (pre)heterosexual males, 346 (pre)homosexual males, and 212 (pre)homosexual females (hereafter referred to as heterosexual males, homosexual males, and homosexual females, respectively) (*N = *768). All probands were patients referred to a Child and Adolescent Gender Identity Service. Probands consisted of consecutive cases who passed through all of the following exclusion criteria: had a diagnosis of a co-occurring disorder of sex development or endocrine disorder, were a twin or triplet, or were adopted or a foster child and accurate information about the number, age, and sex of siblings at the time of initial assessment could not be obtained. In addition, the sample of homosexual males was independent of those reported in Blanchard et al. [22] and Zucker et al. [24].

Probands were referred to this service because of persistent cross-gender behavior and/or identification, transvestic fetishism, or reasons related to homosexual sexual orientation. All probands 12 years of age and younger who were referred for Gender Dysphoria were classified as homosexual in relation to birth sex because follow-up studies have shown that same-sex attraction is the most probable sexual orientation outcome for these children [26]–[29]. (Note: A minority of the gender dysphoric children in our sample will exhibit a heterosexual sexual orientation in adulthood. If heterosexual and homosexual groups differ for the sibship variables of interest, then classifying heterosexual probands as homosexual can only make our heterosexual and homosexual groups more similar. Thus, any significant differences identified between heterosexual and homosexual groups will exist despite, not because of, the inclusion of heterosexual probands in the homosexual groups.).

All individuals referred for homosexual sexual orientation were classified as homosexual. All males referred for transvestic fetishism were classified as heterosexual because transvestic fetishism is characterized by predominant sexual attraction to females [41]. For the majority of adolescents (i.e., 13 years of age and older) referred for Gender Dysphoria, sexual orientation was classified using items from the Erotic Response and Orientation Scale (EROS) and the Sexual History Questionnaire (SHQ) [41]. These questionnaires consisted of items pertaining to the frequency of past attraction toward and sexual activity with males and females, with equal numbers of items pertaining to each sex. Example items from the EROS include “How often have you had any sexual feelings, even the slightest, while looking at a boy?” and “How often have you had any sexual feelings, even the slightest, while looking at a girl?” The response scale was 1 (*none*) to 5 (*almost every day*). Example items from the SHQ include “How many girls have you touched on the naked breasts since the age of 13?” and “Since the age of 13, how many boys have you touched on their private parts with your hands?” Each item was rated on a 5-point scale from 1 (*none* or *never*) to 5 (11 *or more*). Responses were averaged for the same-sex items and opposite-sex items, respectively, and the difference was calculated (same-sex minus opposite-sex) on each questionnaire separately. The scores from each scale were then summed for a total sexual orientation score. Probands with positive scores were classified as homosexual while those with negative scores were classified as heterosexual. The mean (SD) total sexual orientation scores were −1.74 (1.63) for heterosexual males, 2.45 (1.53) for homosexual males, and 2.46 (1.78) for homosexual females. In the event that a proband had equal ratings for same-sex and opposite-sex sexual partner preference (most often because the proband reported no history of sexual attraction or behavior), the proband was classified as heterosexual; however, if information obtained in the course of clinical assessment and treatment provided a clear indication of a preference for the same sex, the proband was classified as homosexual. For three adolescent males, sexual orientation classifications indicated by the questionnaire data were overturned based on information obtained in the course of clinical assessment and treatment. For a minority of adolescents, no questionnaire data were available and sexual orientation classifications were made based on clinical information. Table 1 shows the number of participants according to the basis of referral and sexual orientation classification by group.

**Table 1:** Number of participants according to basis for referral.

Basis for Referral	Heterosexual Males (*n* = 210)	Homosexual Males (*n* = 346)	Homosexual Females (*n* = 212)
Transvestic Fetishism	151	0	0
Homosexuality	0	37	18
Gender Dysphoria (Children)^a^	0	236	91
Gender Dysphoria (Adolescents)			
Clinical Judgment (No SHQ or EROS available)	3	14	14
SHQ and EROS	54	56	85
SHQ and EROS available, but classification made on clinical grounds	2	3	4

a Gender dysphoric children were assigned to the homosexual groups because previous research has shown that same-sex sexual preference is the most likely sexual orientation outcome for these children.

### Measures

Details about the family demographic background of the probands were gathered during interviews at the time of initial clinical assessment. For the purposes of the present study, the numbers of total siblings, older brothers, older sisters, younger brothers, and younger sisters were recorded for each proband. The number of total siblings is simply the sum of siblings of the four types and does not include the proband. Only siblings who were alive at birth were considered. Because the maternal immune hypothesis is specific to siblings who share the same mother, only full and maternal half siblings were included. Adopted, foster, step, and paternal half siblings were ignored. Each proband’s age (in years) was also recorded.

### Data Availability

To acquire data used in this study, please contact the corresponding author.

## Results

### Descriptive Statistics

Descriptive statistics pertaining to probands’ ages and numbers of total siblings, older brothers, older sisters, younger brothers, and younger sisters as a function of group are shown in Table 2. Groups were first compared for age and total numbers of siblings using one-way analysis of variance (ANOVA). There were significant main effects of group for age, *F*(2, 765) = 84.97, *p*<.001, as well as for total number of siblings, *F*(2, 765) = 3.93, *p = *.02. Post hoc Scheffé multiple range tests showed that all groups differed significantly from one another for age at the *p*<.001 level, and homosexual females had significantly fewer siblings compared to heterosexual males (*p = *.02). Due to group differences in total numbers of siblings, as per Blanchard’s [38] recommendation, the total number of siblings was controlled in subsequent analyses by converting number of siblings in each category to proportion of siblings in each category. Thus, four new variables were calculated: proportion of older brothers (number of older brothers/total number of siblings), proportion of older sisters (number of older sisters/total number of siblings), proportion of younger brothers (number of younger brothers/total number of siblings), and proportion of younger sisters (number of younger sisters/total number of siblings). Such proportions could not be calculated for only children and only children were therefore considered in separate analyses presented here. (Note: proportions of siblings were also analyzed following Blanchard’s [38] method for calculating proportions of older and younger brothers and sisters for all probands, including only children. Using this alternate method did not impact the significance of sibling category effects reported here; however, as explained by Blanchard [38], the odds ratios associated with sibling category effects cannot be interpreted when using this alternate method. As such, we have presented analyses for which the odds ratios associated with the sibling category effects can be meaningfully interpreted.).

**Table 2:** Descriptive statistics for age and numbers of siblings.

Variable	Heterosexual Males(*n* = 210)	Homosexual Males(*n* = 346)	Homosexual Females(*n* = 212)
	M (SD)	M (SD)	M (SD)
Age	14.38 (2.55)	9.61 (4.87)	12.33 (4.57)
Total Siblings	1.53 (1.08)	1.38 (1.03)	1.24 (1.07)
Older Brothers	.35 (.66)	.42 (.67)	.29 (.58)
Older Sisters	.31 (.61)	.31 (.65)	.40 (.65)
Younger Brothers	.46 (.71)	.35 (.57)	.26 (.61)
Younger Sisters	.40 (.64)	.29 (.53)	.29 (.53)

Descriptive statistics pertaining to the proportions of siblings in each category are shown in Table 3. Converting numbers of siblings in each category to proportions did not alter the overall sibship profiles of the various groups. Yet, doing so was an effective means of controlling for total number of siblings as evidenced by the near-zero correlations between total number of siblings and these proportions (all Pearson’s *r*’s were between −.06 and +.06).

**Table 3:** Proportions of older brothers, older sisters, younger brothers, and younger sisters among probands with at least one sibling.

Variable	Heterosexual Males(*n* = 186)	Homosexual Males(*n* = 292)	Homosexual Females(*n* = 160)
	M (SD)	M (SD)	M (SD)
Proportion of Older Brothers	.21 (.35)	.31 (.42)	.24 (.38)
Proportion of Older Sisters	.20 (.36)	.20 (.36)	.31 (.41)
Proportion of Younger Brothers	.31 (.41)	.27 (.39)	.20 (.36)
Proportion of Younger Sisters	.28 (.39)	.22 (.36)	.26 (.40)

### Only Children

For homosexual females, 24.5% (52 of 212) were only children compared to 15.6% (54 of 346) of homosexual males and 11.4% (24 of 210) of heterosexual males, χ^2^(2) = 13.66, *p = *.001. Given the parallel group differences in age and frequency of only children, a backward stepwise binary logistic regression analysis was conducted in which only child status was the criterion variable, and age and group were predictors with heterosexual males being designated as a reference group. Age was not significantly associated with only child status, *B (SE) = *.03 (.02). There was no significant difference between the two male groups for only child status, *B (SE) = *.36 (.26). Homosexual females were significantly more likely to be only children compared to heterosexual males, *B (SE) = *.92 (.27), *p* = .001, and this difference was associated with an odds ratio of 2.52.

### Birth Order

Further analyses of sibship composition included 186 heterosexual males, 292 homosexual males, and 160 homosexual females with at least one sibling. The fraternal birth order effect was examined using backward stepwise multinomial regression analysis in which group membership was regressed on the following variables: age, total number of siblings, proportion of older brothers, proportion of older sisters, proportion of younger brothers, and proportion of younger sisters. Heterosexual males were the reference group. Using the backward elimination procedure, variables that did not contribute to the prediction of group membership were removed from the regression equation, starting with the least predictive while variables that did contribute to the prediction of group membership were retained in the final model (Table 4). Table 5 shows the likelihood ratio tests for variables in the final model. Table 6 shows the parameter estimates for the final multinomial equation, which describes the pattern of group differences.

**Table 4:** Summary of the steps in constructing the multinomial regression model using the backward elimination method.

Model	Action	Effect(s)	−2 Log Likelihood	χ^2^ for removal^a^	*df*	*p*
0	Entered	All effects	794.42			
1	Removed	Proportion of Younger Brothers	794.42	0^b^	0^b^	
2	Removed	Total Number of Siblings	795.26	.84	2	.66
3	Removed	Proportion of Younger Sisters	798.76	3.50	2	.17

a The χ^2^ for removal is based on the likelihood ratio test.

b Because each proband’s proportions of older brothers, older sisters, younger brothers, and younger sisters is necessarily summed to 1.00, these proportions were perfectly multicollinear. To reduce the multicollinearity, the computational algorithm of the SPSS multinomial logistic regression program eliminated the proportion of younger brothers from the set of predictor variables.

**Table 5:** Likelihood ratio tests for variables in the final multinomial equation.

Effect	−2 log likelihood of reduced model	χ^2^	*df*	*p*
Intercept	928.98	130.22	2	<.001
Proportion of Older Sisters	807.00	8.24	2	.016
Proportion of Older Brothers	803.44	4.69	2	.096
Age	931.84	133.09	2	<.001

Note: The χ^2^ statistic is the difference in −2 log likelihoods between the final model and a reduced model. The reduced model is formed by omitting an effect from the final model. The null hypothesis is that all parameters of that effect are 0.

**Table 6:** Parameter estimates for the final multinomial equation.

Diagnostic Groups^a^	*B*	*SE*	Wald	*df*	*p*	Odds Ratio
Homosexual Males						
Intercept	3.56	.39	83.99	1	<.001	
Age	−.27	.03	96.42	1	<.001	.77
Proportion of Older Brothers	.61	.29	4.59	1	.032	1.84
Proportion of Older Sisters	−.02	.30	<.01	1	.958	.98
Homosexual Females						
Intercept	1.47	.43	11.94	1	.001	
Age	−.14	.03	23.90	1	<.001	.87
Proportion of Older Brothers	.37	.31	1.40	1	.236	1.45
Proportion of Older Sisters	.70	.30	5.36	1	.021	2.01

a The reference category is heterosexual males.

Compared to heterosexual males, homosexual males were significantly younger and had a significantly greater proportion of older brothers in their sibships. The odds ratio associated with this older brother effect was 1.84. Compared to heterosexual males, homosexual females were significantly younger and had a significantly greater proportion of older sisters in their sibships. The odds ratio associated with this older sister effect was 2.01.

### Sibling Sex Ratio

In addition to the sibship composition parameters of the three groups being compared to one another, they were also compared to well-established population norms. Specifically, we examined the sibling sex ratio, which is the ratio of brothers to sisters collectively reported for a given group of probands. In Western human populations, the ratio of male to female live births is stable at 106∶100 [30], [31]. The ratio of brothers to sisters reported for a group of probands drawn at random from the general population should, therefore, approach 106 brothers to 100 sisters.

Table 7 shows the data pertaining to the overall sibling sex ratio, older sibling sex ratio, and younger sibling sex ratio by group. These ratios were compared to the population sex ratio using the *z*-approximation to the binomial test. For heterosexual males, no significant deviations from the population value were observed. Homosexual males had a significantly elevated overall sibling sex ratio, *z* = 2.01, *p* = .044, but this pattern was driven by a significantly elevated older sibling sex ratio, *z* = 1.96, *p* = .048. Homosexual females had a significantly lower than expected overall sibling sex ratio, *z* = −2.16, *p* = .031, but this pattern was driven by a significantly lower than expected older sibling sex ratio, *z* = −2.18, *p* = .028.

**Table 7:** Sibling sex ratio by group.

Group	Brothers	Sisters	Overall Sex Ratio	Older Sibling Sex Ratio	Younger Sibling Sex Ratio
Heterosexual Males	170 (OB 73, YB 97)	151 (OS 66, YS 85)	112.6∶100	110.6∶100	114.1∶100
Homosexual Males	267 (OB 147, YB 120)	209 (OS 108, YS 101)	127.8∶100*	136.1∶100*	118.8∶100
Homosexual Females	117 (OB 62, YB 55)	144 (OS 84, YS 62)	81.3∶100*	73.8∶100*	88.7∶100

Note: Older Brothers (OB), Older Sisters (OS), Younger Brothers (YB), Younger Sisters (YS).

*p<.05.

## Discussion

The sample employed in the current study improved upon those of previous similar studies examining birth order and sibling sex ratio in gender dysphoric youth samples in certain respects. Previous similar studies [17], [22]–[24], [35] compared gender dysphoric probands, the majority of whom were likely to exhibit homosexuality in adulthood, to clinical or non-clinical control samples that were presumed to be mostly heterosexual in adulthood. In contrast, the present study utilized a sample referred to the same Gender Identity Service and, therefore, was better able to determine the sexual orientations of probands in the various participant groups. Heterosexual male probands’ sexual orientations were verified in the course of clinical assessment and treatment. The homosexual groups consisted of gender dysphoric children who were likely to exhibit homosexuality in adulthood, adolescents who were referred for issues related to homosexuality, and gender dysphoric adolescents whose same-sex sexual orientation was verified in the course of clinical assessment and treatment. In terms of sample size, compared to previous studies, the present study provided the first sample of heterosexual gender-referred youth, the second largest sample of homosexual male youth, and the largest sample of homosexual female youth.

The primary aim of the present study was to evaluate whether the fraternal birth order effect could be detected in the current sample. Many, but not all, previous studies have examined whether a preponderance of older brothers is associated with male homosexuality by directly assessing numbers of older and younger brothers and sisters. Due to group differences in sibship size, however, total number of siblings was controlled. Group differences in the proportions of sibships that consisted of older and younger brothers and sisters were examined instead. The sibships of homosexual males had significantly greater proportions of older brothers compared to those of heterosexual males. This effect was independent of group differences in age. Based on the odds ratio when comparing homosexual to heterosexual males, the odds of homosexuality increase by approximately 84% as one moves from sibships containing no older brothers to those containing older brothers only.

The analyses of sibling sex ratios also indicated a unique preponderance of older brothers among homosexual males. The observed sibling sex ratios were compared to the population ratio of live male to female births (i.e., 106∶100). For heterosexual males, the overall, older, and younger sibling sex ratios did not differ significantly from the population value. For homosexual males, the overall sibling sex ratio was significantly elevated relative to the population value; however, additional analyses showed that only the older, not the younger, sibling sex ratio was significantly higher than expected. Thus, the elevated sibling sex ratio was primarily owing to the fact that homosexual males had a significantly higher number of older brothers to sisters than one would expect from a group of individuals drawn from the population at random. In combination with the finding that homosexual males’ sibships were comprised of significantly greater proportions of older brothers, this significantly elevated older sibling sex ratio among homosexual males demonstrates the existence of the fraternal birth order effect in the present sample.

Compared to previous studies in male youth samples, the present findings provided more straightforward evidence for a fraternal birth order effect. In their small Dutch sample, Blanchard et al. were only able to demonstrate that gender dysphoric male youth were significantly later born relative to clinical controls [17]. Blanchard et al. [22] and Zucker et al. [24] were able to show that gender dysphoric males had significantly elevated sibling sex ratios and were significantly later born; however, these studies were unable to clearly show that that these effects were owing to older brothers in particular. In Schagen et al., although gender dysphoric males had significantly greater numbers of older brothers than controls, they also had significantly fewer older and younger sisters than controls. In addition, both the older and younger sibling sex ratios were significantly elevated in Schagen et al. [23]. Thus, none of the prior studies that utilized gender dysphoric youth samples showed a clear and unique relationship between male sexual orientation and the presence of older brothers. The present study, in contrast, demonstrated that only older brothers were predictive of male sexual orientation and that only the older sibling sex ratio was significantly elevated in homosexual male youth.

With respect to same-sex attracted gender dysphoric adult males or their closest non-Western equivalents, late birth order has been documented in numerous studies [17]–[19], [42]–[47]. In a subset of these studies, elevated sibling sex ratios have been observed among these males as well [17], [19], [42], but not among older siblings specifically. Importantly, elevated sibling sex ratio and late birth order have not been observed among nonhomosexual transsexuals [17], [19], [42], [48], yet only three studies of transgendered or transsexual adult males have examined group differences in older brothers directly while controlling for other sibling category effects [18], [45], [48]. Of these, two Samoan studies found sibling category effects other than the older brother effect, utilized gender-typical male comparison groups, and did not find sibling sex ratios that were elevated compared to the population parameter [18], [45]. The third study found that homosexual, compared to nonhomosexual, male-to-female transsexuals had significantly more older brothers, but failed to find an elevated sibling sex ratio [48]. In contrast to this adult literature, homosexual males in the present youth sample showed both a significantly elevated older sibling sex ratio and a preponderance of older brothers compared to heterosexual males referred to the same clinic while controlling for other sibling category effects. Thus, the current study stands out in the context of similar adult literature as well in that it was better able to demonstrate the key aspects of the fraternal birth order effect among gender-atypical male samples.

The data presented here on male children and adolescents were consistent with the maternal immune hypothesis. As Blanchard and Klassen noted, thoroughly assessing this hypothesis requires consideration of females as well [5]. Any indication of the fraternal birth order effect in female sexual orientation would challenge the maternal immune hypothesis, which posits fetal production of male-specific proteins as an underlying cause for this effect. In the current sample, homosexual females were significantly more likely to be only children and had a significant preponderance of older sisters. This latter finding was reflected in two ways. First, homosexual females’ sibships consisted of greater proportions of older sisters compared to heterosexual males. Second, the older sibling sex ratio of homosexual females was significantly lower than that expected for this population parameter. Neither of these findings is indicative of a fraternal birth order effect. Thus, the overall findings of the present study were in line with the maternal immune hypothesis.

The patterns documented here for homosexual females are intriguing. When considered alongside the handful of relevant studies, they help point toward potentially stable patterns in the sibships of homosexual females, especially those from gender-referred samples. The current study was the first to identify a significantly lower than expected older sibling sex ratio among homosexual females. Yet, a number of previous studies reported that female homosexuality and/or gender dysphoria is associated with a lower ratio of brothers to sisters more generally. Lang found a significantly lower than expected sibling sex ratio in a lesbian sample [49]; however, similar effects were not found in three other lesbian samples that had sibling sex ratios ranging from 97–121∶100 (for review, see [1]) while another study reported a ratio of 113∶100 for right-handed homosexual females and a significantly elevated ratio of 186∶100 among non-right-handed homosexual females [50]. In adult female-to-male homosexual transsexuals, Green found a significantly low sibling sex ratio of 79∶100 [48], although Blanchard and Sheridan [42] and Gómez-Gil et al. [19] did not with their respective reported sibling sex ratios of 95∶100 and 99∶100. In gender dysphoric girls, Schagen et al. found a low sibling sex ratio of 77∶100 and argued that the lack of statistical significance was due to small sample size [23]. This argument may be correct in conjecturing that this sex ratio would remain stable and achieve statistical significance with an increased sample given its similarity to the significant ratios of 74∶100 observed in the present study and that of 79∶100 observed by Green [48]. Overall, these studies provide mixed results and it is unclear what factors might be accounting for differences across studies. Furthermore, if these findings of lower sibling sex ratios are not spurious, it is unclear whether these sibling sex ratio patterns might be characteristic of homosexual females in general or are specific to gender dysphoric homosexual females. In any case, the growing number of studies that are converging on this same observation warrants future research regarding this issue and may help in identifying unique factors involved in the development of female sexual orientation and gender identity.

The present findings regarding only children are also consistent with the available previous literature. In total, 24.5% of the probands in our predominantly gender dysphoric female sample were only children. This percentage was significantly greater than the two male groups, and the significant difference remained even when controlling for age. Although we were unable to provide a comparison group of gender-referred heterosexual female youth (in other words, biological females who wish to become gay males), this percentage was consistent with that observed by Schagen et al. [23]. In their sample of Dutch gender dysphoric girls, 29.5% were only children, which was significantly elevated compared to 12.7% of girls in their sample of non-clinical control females. It remains unclear, however, whether the high percentage of only children among gender dysphoric girls is characteristic of clinic-referred girls in general. Therefore, future research comparing proportions of only children among gender-referred vs. other clinic-referred girls is necessary.

Blanchard proposed a maternal-fetal interaction differing from that postulated by the maternal immune hypothesis that might account for these only children findings [51]. Specifically, mothers who are prone to producing immune responses to non-sex-specific fetal antigens might produce antibodies that can affect fetal neural development, impacting traits such as sexual orientation–and, in the present case, gender identity as well. These same maternal immune reactions could create difficulty in initiating or maintaining pregnancy, thus accounting for why certain groups, such as gender dysphoric homosexual females, are more likely to be only children. In support of this hypothesis, mothers of homosexual females reported a higher proportion of pregnancies terminating before six months compared to the mothers of heterosexual females and heterosexual and homosexual males [52]. Future research is necessary to discern the extent to which the findings on females reported here are replicable. If they are indeed replicable, then research is also needed to discern whether Blanchard’s hypothesis [51] or some as of yet to be specified factor(s) are capable of accounting for this phenomenon. Furthermore, if this type of maternal-fetal interaction and the one proposed by the maternal immune hypothesis both exist, there may be two distinct sibship type clusters among homosexual males (i.e., a cluster of only children and a cluster with late fraternal birth order). Future research should also discern whether such is the case.

Although the present findings are largely consistent with previous literature on birth order and sibling sex ratio among gender dysphoric youth and adults, they departed from two previous studies utilizing larger representative samples of U.S. adolescents in important respects [36], [37]. These studies did not find the fraternal birth order effect, and contrary to the present study and other studies, Francis reported that his homosexual female group had significantly fewer, rather than significantly more, sisters. As highlighted above, certain methodological factors other than recruitment strategy may account for these discrepancies. Interestingly, however, the study by Francis indicated that homosexual females had significantly fewer siblings, which might relate to the greater frequency of only children found among gender dysphoric girls in the present study and in that of Schagen et al. [23]. To evaluate whether patterns found in gender dysphoric youth apply to youth more generally, future research addressing these various methodological issues in adolescent samples from the general population will be necessary.

In sum, the present study provided clear evidence of the fraternal birth order effect in a youth sample from a Gender Identity Service and is, therefore, consistent with the maternal immune hypothesis. The results were consistent with those of previous studies examining samples consisting of gender dysphoric youth or adults. Homosexual, compared to heterosexual, males showed a significant preponderance of older brothers and a significantly elevated older sibling sex ratio. Homosexual females did not show any sibship patterns consistent with the fraternal birth order effect, but did show a significant preponderance of older sisters and a greater likelihood of being only children. These findings were consistent with the small literature on the sibship composition of females in relation to sexual orientation and gender identity. Future research is needed to discern the extent to which the latter findings are replicable. Doing so will aid in understanding the development of female sexual orientation and gender identity.
